# Association between relative fat mass and cognitive impairment in older adults: A cross-sectional study using NHANES 2011–2014 data

**DOI:** 10.1097/MD.0000000000049621

**Published:** 2026-07-10

**Authors:** Zhihui Wu, Lili Zhang, Weijiao Feng, Yan Huang

**Affiliations:** a Department of Neurology, Affiliated Hospital of Jiangnan University, Wuxi, China; b Department of Internal Medicine, Affiliated Hospital of Jiangnan University, Wuxi, China; c Department of Neurosurgery, Suzhou Kowloon Hospital Shanghai Jiao Tong University School of Medicine, Suzhou, China.

**Keywords:** cognitive impairment, hypertension, NHANES, older adults, physical activity, relative fat mass

## Abstract

Relative fat mass (RFM), derived from height and waist circumference, may better reflect overall and central adiposity than body mass index. This study examined the association between RFM and cognitive impairment among older adults in the United States. We conducted a cross-sectional analysis using National Health and Nutrition Examination Survey 2011–2014 data, including 2730 participants aged ≥ 60 years with valid anthropometric data, cognitive assessment data, and covariate information required for the primary analysis. Cognitive function was evaluated using the Consortium to Establish a Registry for Alzheimer Disease (word learning & delayed recall tests), animal fluency, and digit symbol substitution tests. Cognitive impairment was defined as performance below the education-specific 25th percentile of a standardized global cognition *z*-score. Weighted logistic regression models estimated associations between RFM and cognitive impairment, adjusting for demographic, socioeconomic, lifestyle, and clinical factors. Nonlinearity was tested using restricted cubic spline and two-piecewise regression analyses. In the base-adjusted model, RFM was not significantly associated with cognitive impairment. After full adjustment for demographic, socioeconomic, lifestyle, dietary, and clinical factors, higher RFM was associated with increased odds of cognitive impairment (odds ratio = 1.05; 95% confidence interval: 1.02–1.09). Sensitivity analyses excluding participants with very high body mass index, trimming RFM outliers, modeling log-transformed RFM, and using waist circumference as an alternative adiposity measure yielded directionally consistent results. The relationship was linear (*P*_overall = .042; *P*_nonlinear = .253), with stronger associations in participants aged < 70 years, and those with hypertension or prior stroke. Hypertension (10%) and physical inactivity (9.4%) partially mediated this relationship, whereas depression and diabetes showed minimal effects. Higher RFM was associated with greater odds of test-based cognitive impairment among United States older adults after multivariable adjustment. The observed associations involving hypertension and physical activity status were exploratory and should not be interpreted as causal. Future longitudinal studies are needed to clarify temporality and evaluate the potential clinical utility of RFM in cognitive risk assessment.

## 1. Introduction

Cognitive impairment is a broad construct referring to reduced performance in 1 or more cognitive domains, such as memory, attention, executive function, verbal fluency, or processing speed, that may interfere with independence and activities of daily living. In this study, cognitive impairment refers to low performance on a standardized composite cognitive score derived from National Health and Nutrition Examination Survey (NHANES) cognitive tests, rather than a clinical diagnosis of mild cognitive impairment (MCI) or dementia. MCI is a clinically defined syndrome that represents an intermediate state between normal cognitive aging and dementia, including Alzheimer disease.^[1]^ Recent meta-analyses suggest that MCI affects approximately 19 to 21% of adults older than 50 years worldwide.^[2]^ Although cognitive impairment is also highly prevalent among older adults in nursing homes and long-term care facilities,^[3]^ the present study focuses on noninstitutionalized older adults represented in NHANES. Individuals with MCI have a substantially higher risk of progressing to dementia, with annual conversion rates of approximately 10 to 15%.^[4]^

Cognitive impairment affects not only cognitive function but also functional independence, disability risk, risk of nursing home admission, and mortality. On a social scale, it places considerable economic and caregiving demand and threatens the viability of health care systems.^[5]^ While new interventions (vascular risk reduction, physical exercise, diet, cognitive stimulation and early detection) may have the potential to delay disease progression, there is currently no curative treatment. Additional studies are required to elucidate the mechanisms involved, enhance screening and provide scalable prevention.

Many observational cross-sectional and longitudinal studies have associated elevated body mass index (BMI), calculated as weight in kilograms divided by height in meters squared, and central adiposity with worse cognitive performance across multiple domains.^[6]^ Nonetheless, traditional anthropometric measures such as BMI and waist circumference (WC), a simple measure of abdominal adiposity, have important limitations when used to evaluate adiposity in older adults. BMI cannot differentiate between lean mass and fat mass and may underestimate adiposity in individuals with sarcopenic obesity or age-related muscle loss; WC, although useful for assessing abdominal adiposity, does not account for height or sex-specific differences in body composition.^[7]^

To address these limitations, relative fat mass (RFM) has been proposed as a simple anthropometric index for estimating body fat percentage. Unlike BMI, RFM incorporates height and WC. Unlike WC alone, RFM standardizes abdominal size by height and uses a sex-specific equation. Previous validation studies have shown that RFM correlates more closely with body fat percentage estimated by dual-energy X-ray absorptiometry than BMI or WC alone. Therefore, RFM may provide additional clinical value as a low-cost and scalable marker of adiposity-related cardiometabolic and neurovascular risk, particularly in population-based studies and routine clinical settings.^[8]^

The relationship between adiposity and cognitive impairment has international relevance, as population aging and obesity are increasing global public health challenges. However, adiposity–cognition associations may differ across populations because of variations in body composition, cardiometabolic risk, lifestyle, and cognitive assessment methods. Evidence from China and other cohorts suggests that BMI and WC may show heterogeneous associations with cognitive outcomes, supporting the need to evaluate alternative indicators such as RFM in nationally representative samples.

Recent studies have linked higher RFM with cardiometabolic and vascular abnormalities, including hypertension, dyslipidemia, insulin resistance, arterial stiffness, carotid atherosclerosis, and subclinical vascular inflammation.^[8,9]^ Population-based studies have also associated elevated RFM with stroke and coronary heart disease (CHD), supporting its potential value as a marker of vascular burden.^[10,11]^ These metabolic and vascular conditions are closely related to cognitive decline in later life. Compared with BMI and WC, RFM may better capture the metabolic and vascular burden associated with central adiposity. Because it can be calculated from simple anthropometric measures, RFM may be useful in large epidemiological studies such as NHANES.

We performed a cross-sectional study using NHANES 2011–2014 data among United States (US) adults aged ≥ 60 years to examine the association between RFM and cognitive impairment. By using a nationally representative sample of noninstitutionalized older adults, this study aimed to provide evidence on whether RFM, as an alternative adiposity measure, is associated with cognitive impairment after adjustment for demographic, behavioral, and cardiometabolic factors.

## 2. Materials and methods

### 2.1. Study population

**Figure 1.**
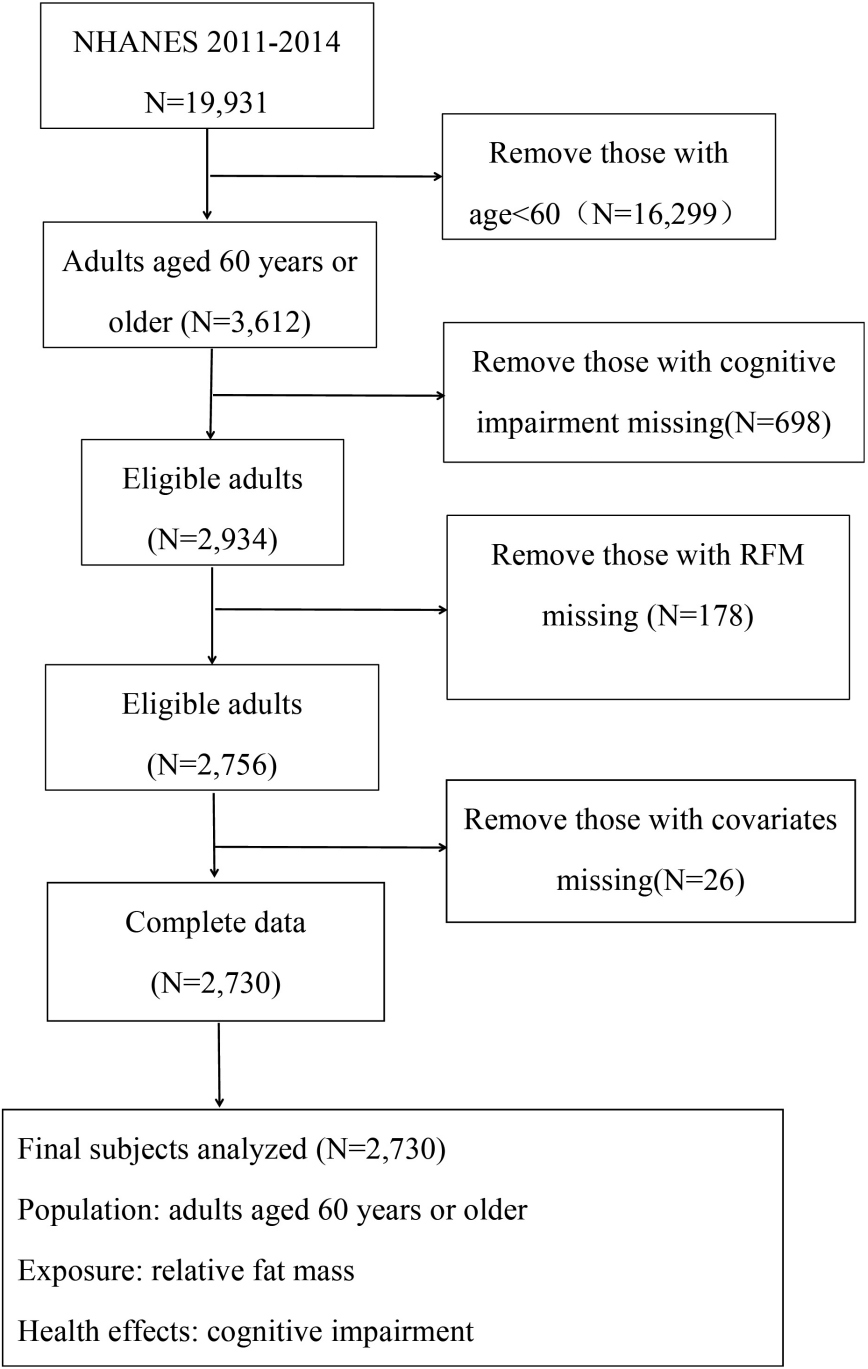
Flow diagram of the inclusion criteria and exclusion criteria. NHANES = National Health and Nutrition Examination Survey, RFM = relative fat mass.

We used data from NHANES 2011–2012 and 2013–2014 in this cross-sectional analysis. NHANES is a nationally representative survey of the civilian, noninstitutionalized US population conducted by the Centers for Disease Control and Prevention’s National Center for Health Statistics using a complex multistage sampling design. NHANES accrues standardized interview data, physical examination, and laboratory studies in Mobile Examination Centers.^[12]^ From an initial 19,931 participants, we restricted the sample to adults aged ≥ 60 years (n = 3612) who completed the cognitive testing battery and had valid anthropometric data for calculating RFM. We then excluded participants without sufficient cognitive data to define cognitive impairment (n = 698), those missing height, WC, or sex required for calculating RFM (n = 178), and those missing essential covariates included in the fully adjusted model (n = 26). The final complete-case analytic cohort included 2730 older adults for examining the cross-sectional association between RFM and cognitive impairment (Fig. 1). All NHANES procedures were approved by the National Center for Health Statistics Institutional Review Board, and written informed consent was obtained from all participants.

### 2.2. Evaluation of cognitive impairment

Cognitive performance (NHANES 2011–2014; participants aged ≥ 60 years) was evaluated with 3 established tests: the Consortium to Establish a Registry for Alzheimer Disease (Word Learning & Delayed Recall tests – CERAD) Word Learning subtests – 3 immediate learning trials and 1 delayed recall trial – capturing memory; the Animal Fluency test, indexing categorical verbal fluency as an aspect of executive function (1-minute animal naming, 1 point per correct response); and the digit symbol substitution test (DSST) from the Wechsler Adult Intelligence Scale, reflecting processing speed, sustained attention, and working memory. These instruments and their NHANES administration have been widely used in recent work.

For analysis, raw scores from the available cognitive tests were standardized within the analytic sample as *z*-scores. A memory score was calculated as the mean of the CERAD immediate-learning and delayed-recall *z*-scores when both were available; otherwise, the available component was used. A global cognition score was then calculated as the mean of the available domain-specific *z*-scores, including memory, verbal fluency, and processing speed. Higher scores indicated better cognitive performance. Cognitive impairment was defined using an education-specific percentile-based threshold. Education level was classified using DMDEDUC2, the NHANES adult education-level variable that records the highest grade or degree completed by participants aged 20 years or older. The DMDEDUC2 categories include <9th grade, 9th–11th grade, high school graduate/general educational development or equivalent, some college or AA degree, and college graduate or above. Within each DMDEDUC2 education stratum, participants with a global cognition *z*-score at or below the 25th percentile were classified as having cognitive impairment, and all others were classified as not having cognitive impairment. The 25th percentile cutoff was selected because there is no universally accepted clinical cutoff for defining cognitive impairment using the NHANES CERAD, animal fluency, and DSST tests. Prior NHANES-based studies have commonly used the lowest quartile of cognitive test performance to identify low cognitive performance or cognitive impairment. We used an education-specific cutoff to reduce misclassification related to differences in educational attainment and test-taking background. Therefore, the outcome should be interpreted as test-based cognitive impairment rather than a clinical diagnosis of MCI or dementia.^[12]^

### 2.3. Measurements of RFM

RFM served as the exposure. Anthropometry was obtained in NHANES Mobile Examination Centers by trained staff: WC was taken at the superior lateral border of the right iliac crest at end-expiration with a non-stretch tape, and height was measured using a portable stadiometer. RFM was calculated as 64 − 20 × (height [cm]/waist [cm]) + 12 × sex, where sex = 1 for women and 0 for men. Values were recorded to 2 decimals and analyzed as a continuous variable.^[8]^

### 2.4. Covariates

Guided by prior work and NHANES analytic conventions, potential confounders were prespecified across demographic, socioeconomic, behavioral, anthropometric, and clinical domains. Demographics included age (continuous), sex (male/female), and race/ethnicity using NHANES standard categories. Socioeconomic status was captured by education level (less than high school; high school/general educational development; and more than high school), marital status (married/cohabiting; widowed; divorced/separated; and never married), and the poverty–income ratio (PIR) categorized as ≤ 1.3, 1.3 to 3.5, or > 3.5 of the federal poverty level. Behavioral factors comprised smoking (ever smoked ≥ 100 cigarettes in lifetime with current use derived per NHANES skip patterns), Drinker status was defined as ≥ 12 alcoholic beverages in the last 12 months; otherwise (including never), nondrinker, and physical activity based on the NHANES physical activity questionnaire for adults. Dietary intake was indexed by total energy intake (kcal) from the 24-hour dietary recall (what we eat in America). Anthropometrics included BMI (kg/m^2^) calculated from measured height and weight. Clinical comorbidities – hypertension, diabetes, CHD, and stroke – were ascertained following NHANES interview and medication-use protocols, and depression was derived from the NHANES 9-item depression screener (Patient Health Questionnaire-9) according to established cut points. All covariates (gender, age, race, education level, marital status, PIR, smoking, physical activity, CHD, stroke, total energy intake [kcal], hypertension, BMI, drinking status, diabetes, and depression) were defined using NHANES codebooks to ensure cross-cycle comparability.^[13]^

We assessed multicollinearity among all covariates (gender, age, race, education level, marital status, PIR, smoking, physical activity, CHD, stroke, total energy intake [kcal], hypertension, BMI, drinking status, diabetes, and depression) before model fitting. The final analytic cohort included complete data for all covariates used in the fully adjusted model. No covariate imputation was performed in the primary analyses. Continuous variables used in diagnostic procedures were standardized within the complete-case analytic sample. Variance inflation factors (VIFs) and tolerances (1/VIF) were computed from a model including all covariates, and condition indices were examined via singular value decomposition (Belsley diagnostics). VIFs were uniformly low – max 1.297 (PIR), min 1.002 (diabetes) – with no VIF > 5 or > 10; the maximum condition index was 5.54. These diagnostics indicate no material multicollinearity among the covariates (Supplementary Table S2, Supplemental Digital Content 1).

### 2.5. Statistical analyses

All analyses followed NHANES analytic guidance and incorporated examination weights (WTMEC2YR), strata (SDMVSTRA), and primary sampling units (SDMVPSU) to obtain nationally representative estimates and valid standard errors under the complex multistage design. Continuous variables were summarized as weighted means with standard errors, and categorical variables as weighted proportions. The association between RFM and cognitive impairment (binary outcome) was evaluated with survey-weighted logistic regression, reporting odds ratios (ORs) and 95% confidence intervals (CIs). RFM was modeled as a continuous exposure (per 1-unit increase) and in quartiles using Q1 as the reference. Three prespecified models were fit: Model 0 unadjusted; Model 1 adjusted for age, sex, and race/ethnicity; and Model 2 further adjusted for education level, marital status, PIR, smoking, drinking status, physical activity, hypertension, diabetes, CHD, stroke, depression, BMI, and total energy intake. Potential nonlinearity was examined with restricted cubic splines (RCSs) using knots at the 10th, 50th, and 90th percentiles of RFM; two-piecewise (segmented) logistic regression identified inflection points by maximum likelihood, with likelihood-ratio tests comparing linear versus piecewise fits. Subgroup analyses explored effect modification by sex, age (<70 vs ≥70 years), race/ethnicity, BMI, smoking, alcohol use, diabetes, hypertension, CHD, stroke, PIR, physical activity, and depression; multiplicative interactions were tested via likelihood-ratio tests. Participants with missing exposure, outcome, or essential covariate data were excluded before model fitting; no median imputation was used in the primary analysis. Primary analyses were conducted in R 4.3.0 and EmpowerStats 5.0; figures were prepared in Zstats 1.0 (www.zstats.net). Two-sided *P* < .05 was considered statistically significant.

Prespecified sensitivity analyses included: exclusion of participants with BMI ≥ 95th percentile; exclusion of RFM outliers (1st–99th percentiles); using log-RFM as the exposure; and using WC (*z*-score) as an alternative exposure, with covariate adjustment and weighting held constant across specifications.

Mediation analysis: We examined whether cardiometabolic/behavioral factors mediated the RFM–cognitive impairment association using prespecified single-mediator models for hypertension, depression, physical activity, diabetes, and BMI. Each mediator was analyzed separately with covariate adjustment matching the fully adjusted main model. We estimated the total effect, average direct effect, and average causal mediation effect (ACME = a × b), and calculated the proportion mediated (ACME/total effect). Variables were standardized. Uncertainty was assessed via nonparametric bootstrap (300 replications) to obtain 95% CIs and *P* values. Analyses were implemented in Python 3.11 (statsmodels, numpy), with mediation diagrams used for visualization.

## 3. Results

### 3.1. Characteristics of the participants

In this cross-sectional NHANES sample of older adults, 2730 participants were included, comprising 2045 without cognitive impairment and 685 with cognitive impairment. Table 1 summarizes baseline characteristics by cognitive status. The overall mean age was 69.25 ± 6.72 years; participants with cognitive impairment were older than those without impairment (72.05 ± 6.81 vs 68.32 ± 6.42 years; *P* < .001).

**Table 1:** Baseline characteristics of participants with cognitive impairment or noncognitive impairment.

Characteristics	Total (n = 2730)	Noncognitive impairment (n = 2045)	Cognitive impairment (n = 685)	*P*-value
Age, yr	69.25 ± 6.72	68.32 ± 6.42	72.05 ± 6.81	<.001
Total energy intake	1847.35 ± 780.41	1884.24 ± 788.74	1737.19 ± 744.74	<.001
Gender, n (%)				<.001
Female	1395 (51.10)	1100 (78.85)	295 (21.15)	
Male	1335 (48.90)	945 (70.79)	390 (29.21)	
Education level, n (%)				1.000
Above high school	1411 (51.68)	1057 (74.91)	354 (25.09)	
High school	637 (23.33)	477 (74.88)	160 (25.12)	
Less than high school	682 (24.98)	511 (74.93)	171 (25.07)	
Marital status, n (%)				0.052
Married/Living with Partner	1604 (58.75)	1225 (76.37)	379 (23.63)	
Never married	155 (5.68)	119 (76.77)	36 (23.23)	
Widowed/Divorced/Separated	971 (35.57)	701 (72.19)	270 (27.81)	
Smoking, n (%)				0.756
No	1341 (49.12)	1001 (74.65)	340 (25.35)	
Yes	1389 (50.88)	1044 (75.16)	345 (24.84)	
Physical activity, n (%)				<.001
No	1966 (72.01)	1431 (72.79)	535 (27.21)	
Yes	764 (27.99)	614 (80.37)	150 (19.63)	
Coronary heart disease, n (%)				0.080
No	2488 (91.14)	1875 (75.36)	613 (24.64)	
Yes	242 (8.86)	170 (70.25)	72 (29.75)	
Stroke, n (%)				<.001
No	2557 (93.66)	1945 (76.07)	612 (23.93)	
Yes	173 (6.34)	100 (57.80)	73 (42.20)	
Hypertension, n (%)				<.001
No	1041 (38.13)	822 (78.96)	219 (21.04)	
Yes	1689 (61.87)	1223 (72.41)	466 (27.59)	
Drinking status, n (%)				0.036
No	853 (31.25)	617 (72.33)	236 (27.67)	
Yes	1877 (68.75)	1428 (76.08)	449 (23.92)	
Diabetes, n (%)				0.184
No	2493 (91.32)	1859 (74.57)	634 (25.43)	
Yes	237 (8.68)	186 (78.48)	51 (21.52)	
Depression, n (%)				0.006
No	2479 (90.81)	1875 (75.64)	604 (24.36)	
Yes	251 (9.19)	170 (67.73)	81 (32.27)	
PIR, n (%)				<.001
<1.3	413 (15.13)	283 (68.52)	130 (31.48)	
1.3-3.5	1334 (48.86)	972 (72.86)	362 (27.14)	
≥3.5	983 (36.01)	790 (80.37)	193 (19.63)	
BMI, n (%)				0.044
<25	735 (26.92)	539 (73.33)	196 (26.67)	
25–30	969 (35.49)	710 (73.27)	259 (26.73)	
≥30	1026 (37.58)	796 (77.58)	230 (22.42)	
RFM	37.07 ± 7.95	37.41 ± 7.97	36.05 ± 7.82	<.001
RFM group, n (%)				<.001
Q1	683 (25.02)	490 (71.74)	193 (28.26)	
Q2	682 (24.98)	482 (70.67)	200 (29.33)	
Q3	682 (24.98)	533 (78.15)	149 (21.85)	
Q4	683 (25.02)	540 (79.06)	143 (20.94)	
Race, n (%)				<.001
Mexican American	247 (9.05)	196 (79.35)	51 (20.65)	
Non-Hispanic Asian	228 (8.35)	170 (74.56)	58 (25.44)	
Non-Hispanic Black	641 (23.48)	427 (66.61)	214 (33.39)	
Non-Hispanic White	1290 (47.25)	1018 (78.91)	272 (21.09)	
Other Hispanic	286 (10.48)	206 (72.03)	80 (27.97)	
Other Race – including multi-racial	38 (1.39)	28 (73.68)	10 (26.32)	

BMI = body mass index, PIR = poverty income ratio, RFM = relative fat mass.

Total energy intake was lower among those with impairment (1737.19 ± 744.74 vs 1884.24 ± 788.74 kcal; *P* < .001). Sex distributions differed between groups (*P* < .001): among females, 21.15% were impaired (295/1395) versus 29.21% among males (390/1335). Race/ethnicity also varied markedly (*P* < .001): the proportion impaired was higher among non-Hispanic Black participants (33.39%) and lower among non-Hispanic White participants (21.09%), with intermediate proportions for Mexican American (20.65%), other Hispanic (27.97%), non-Hispanic Asian (25.44%), and other/multi-racial groups (26.32%). Educational attainment did not differ statistically (*P* = 1.000). Marital status distributions trended toward difference without reaching significance (*P* = .052): married/living with partner 23.63% impaired, widowed/divorced/separated 27.81%, and never married 23.23%. Socioeconomic status showed a clear gradient by PIR (*P* < .001): impairment was most prevalent with PIR < 1.3 (31.48%), intermediate with PIR 1.3–3.5 (27.14%), and least prevalent with PIR ≥ 3.5 (19.63%). BMI categories differed modestly (*P* = .044), with impairment proportions of 26.67% (<25 kg/m^2^), 26.73% (25–30 kg/m^2^), and 22.42% (≥30 kg/m^2^). Lifestyle factors were mixed. Smoking status was not associated with impairment (*P* = .756). Regular physical activity was less common among those with impairment (19.63% vs 27.21% impaired among inactive; *P* < .001). Current drinking was associated with a lower prevalence of impairment (23.92% vs 27.67% among nondrinkers; *P* = .036). Regarding comorbidities, stroke and hypertension were more prevalent among participants with impairment (stroke 42.20% vs 23.93% in those without; hypertension 27.59% vs 21.04% in those without hypertension; both *P* < .001), whereas differences were not observed for CHD (*P* = .080) or diabetes (*P* = .184). Self-reported depression was more frequent among those with impairment (32.27% vs 24.36%; *P* = .006).

With respect to the exposure of interest, mean RFM was lower in the impaired group (36.05 ± 7.82) than in the non-impaired group (37.41 ± 7.97; overall 37.07 ± 7.95; *P* < .001). The distribution across RFM quartiles also differed (*P* < .001), with higher impairment in the lower quartiles (Q1: 28.26%; Q2: 29.33%) and lower impairment in the higher quartiles (Q3: 21.85%; Q4: 20.94%). These descriptive comparisons characterize the sample and do not imply directionality or causation.

### 3.2. Association of RFM and cognitive impairment

Table 2 summarizes the associations between RFM and cognitive impairment across progressively adjusted logistic regression models. In the unadjusted model, higher RFM was inversely associated with cognitive impairment (per-unit OR = 0.98; 95% CI: 0.97–0.99; *P* < .0001). After adjustment for age, sex, and race/ethnicity (Model I), the association attenuated to the null (OR = 1.01; 95% CI: 0.99–1.02; *P* = .5437). In the fully adjusted Model II – which additionally accounted for socioeconomic, lifestyle, and clinical covariates (education, marital status, PIR, smoking, physical activity, CHD, stroke, total energy intake, hypertension, BMI, drinking status, diabetes, and depression) – the association changed direction and reached statistical significance (OR = 1.05; 95% CI: 1.02–1.09; *P* = .0056). Results were directionally consistent when RFM was categorized into quartiles using Q1 (11.93–30.69) as the reference. In unadjusted analyses, mid-to-higher quartiles were associated with lower odds of impairment (Q2: OR = 1.01; 95% CI: 0.84–1.22; *P* = .8853; Q3: OR = 0.66; 95% CI: 0.54–0.81; *P* < .0001; Q4: OR = 0.69; 95% CI: 0.56–0.84; *P* = .0002; *P* for trend < .0001). After adjusting for age, sex, and race/ethnicity (Model I), these associations were attenuated and nonsignificant (Q2: OR = 1.09; 95% CI: 0.89–1.33; *P* = .4102; Q3: OR = 0.94; 95% CI: 0.68–1.31; *P* = .7128; Q4: OR = 1.02; 95% CI: 0.71–1.46; *P* = .9289; *P* for trend = .8009). In the fully adjusted Model II, quartile associations remained nonsignificant (Q2: OR = 1.21; 95% CI: 0.95–1.55; *P* = .1237; Q3: OR = 1.18; 95% CI: 0.76–1.81; *P* = .4608; Q4: OR = 1.34; 95% CI: 0.76–2.37; *P* = .3111; *P* for trend = .3084).

**Table 2:** Association between RFM and cognitive impairment.

Exposure	Non-adjusted model	Model I	Model II
RFM	0.98 (0.97–0.99) < 0.0001	1.01 (0.99–1.02) 0.5437	1.05 (1.02–1.09) 0.0056
Q1 (11.93–30.69)	Ref	Ref	Ref
Q2 (30.69–36.87)	1.01 (0.84–1.22) 0.8853	1.09 (0.89–1.33) 0.4102	1.21 (0.95–1.55) 0.1237
Q3 (36.92–43.83)	0.66 (0.54–0.81) < 0.0001	0.94 (0.68–1.31) 0.7128	1.18 (0.76–1.81) 0.4608
Q4 (43.84–57.29)	0.69 (0.56–0.84) 0.0002	1.02 (0.71–1.46) 0.9289	1.34 (0.76–2.37) 0.3111
*P* for trend	<.0001	.8009	.3084

Model 1 adjust for: gender, age, and race. Model 2 adjust for: gender, age, race, education level, marital status, PIR, smoking, physical activity, coronary heart disease, stroke, total energy intake, hypertension, BMI, drinking status, diabetes, and depression.

BMI = body mass index, PIR = poverty income ratio, RFM = relative fat mass.

Taken together, these cross-sectional estimates indicate that crude analyses suggest a lower odds of impairment at higher RFM, but this relationship is fully explained – and reverses direction – after comprehensive adjustment for sociodemographic, behavioral, and clinical factors when RFM is modeled continuously, whereas categorical analyses show no graded trend in the fully adjusted model. These findings underscore the potential for confounding and scale-dependence in the RFM–cognition relationship and do not imply causality.

#### 3.2.1. Sensitivity analyses

We conducted a series of sensitivity analyses to evaluate the robustness of the association between RFM and cognitive impairment. In the base-adjusted model, which included sex, age, and race/ethnicity, higher RFM was not significantly associated with cognitive impairment (OR = 1.01; 95% CI: 0.98–1.04; n = 2730). After full adjustment for education level, marital status, PIR, smoking, physical activity, CHD, stroke, total energy intake, hypertension, BMI, drinking status, diabetes, and depression, higher RFM was associated with increased odds of cognitive impairment (OR = 1.05; 95% CI: 1.02–1.09; n = 2730). This positive association remained consistent after excluding participants with very high BMI (≥95th percentile; OR = 1.10; 95% CI: 1.04–1.18; n = 2592) and after trimming potential RFM outliers at the 1st and 99th percentiles (OR = 1.10; 95% CI: 1.03–1.18; n = 2674). Modeling RFM on the log scale also yielded a positive association, although the estimate was less precise and should not be directly compared with the per-unit RFM estimate because of the different exposure scale (OR = 6.27; 95% CI: 1.24–31.64; n = 2730). As a construct-level check using central adiposity, each 1-standard deviation higher WC was similarly associated with higher odds of cognitive impairment (OR = 1.37; 95% CI: 1.02–1.84; n = 2730; Supplementary Table S1, Supplemental Digital Content 2).

Collectively, these sensitivity analyses suggested that the positive association between RFM and cognitive impairment in the fully adjusted model was generally robust to alternative model specifications, exclusion of participants with extreme BMI, trimming of RFM outliers, log-transformation of RFM, and substitution of RFM with WC. The visual summary in Supplementary Figure S1, Supplemental Digital Content 3 showed that the fully adjusted estimates were above the null line, supporting the robustness of the observed association without implying causality.

### 3.3. RCS regression analysis between RFM and cognitive impairment

**Figure 2.**
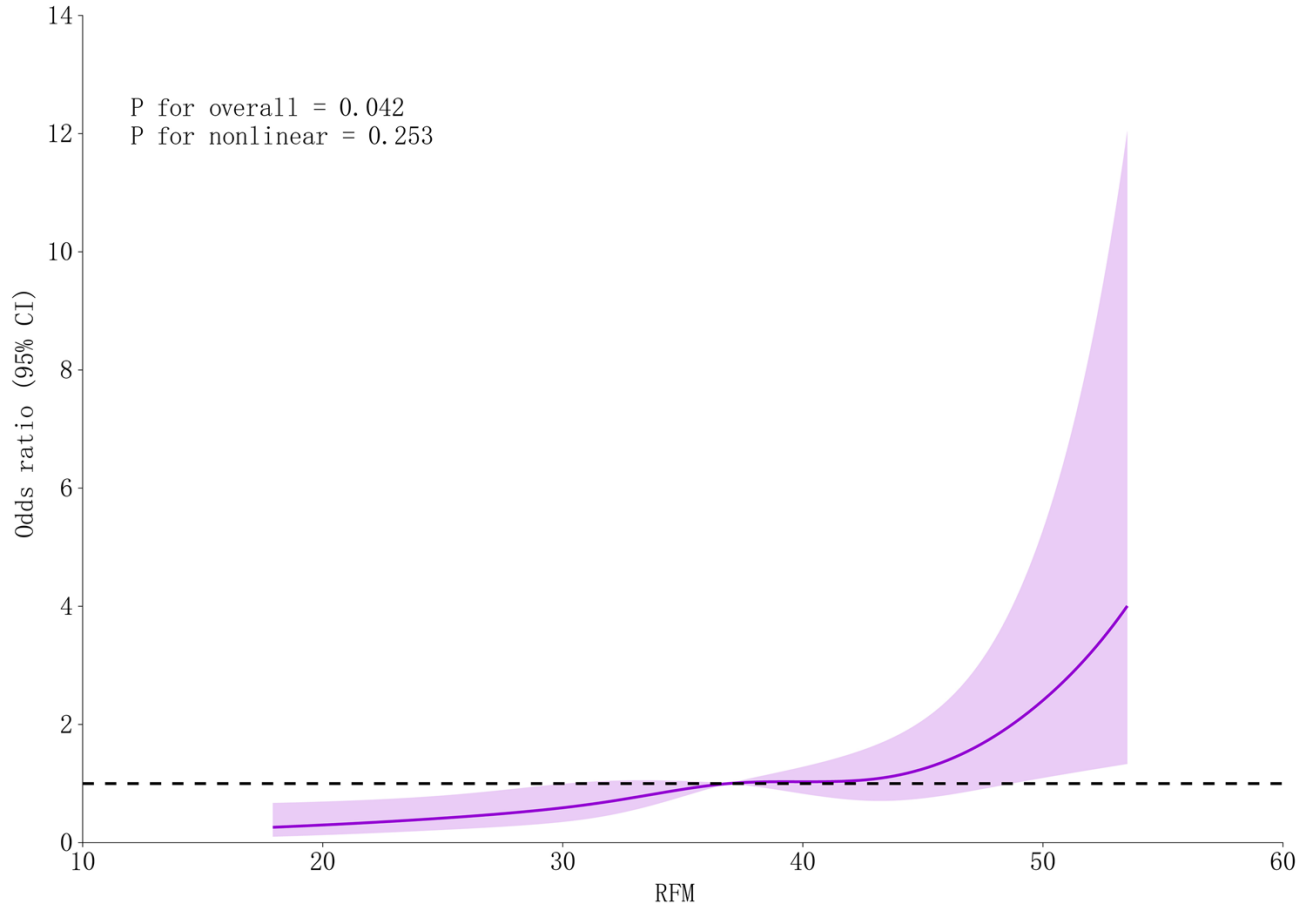
The smoothed curve-fit plot of the dose–response relationship between RFM and cognitive impairment. The associations were adjusted for gender, age, race, education level, marital status, PIR, smoking, physical activity, coronary heart disease, stroke, total energy intake, hypertension, BMI, drinking status, diabetes, and depression. BMI = body mass index, CI = confidence interval, PIR = poverty–income ratio, RFM = relative fat mass.

In the fully adjusted cross-sectional model, the RCS in Figure 2 indicated a significant overall association between RFM and the odds of cognitive impairment (*P* for overall = .042), with no evidence of nonlinearity (*P* for nonlinear = .253). The exposure–response curve rose gradually across mid-range RFM and appeared steeper at higher levels, although uncertainty widened in the upper tail (Fig. 2).

Given the absence of a statistically significant nonlinear pattern, we treated threshold modeling as exploratory. In a sensitivity two-piecewise logistic regression, a data-driven inflection point was identified at RFM = 47.32; below this value, each 1-unit increase in RFM corresponded to 7% higher odds of cognitive impairment (OR = 1.07; 95% CI: 1.02–1.12; *P* = .0076), whereas above 47.32 the slope was steeper (OR = 1.26 per unit; 95% CI: 1.08–1.46; *P* = .0028). The piecewise model modestly improved fit versus a single-slope specification (likelihood ratio test *P* = .021), and the fully adjusted linear model estimated an overall OR of 1.06 per unit RFM (95% CI: 1.01–1.11; *P* = .0153; Table 3).

**Table 3:** Nonlinearity addressing of RFM and cognitive impairment.

Cognitive impairment	OR (95% CI), *P*-value
RFM	
Fitting model by standard logistic regression	1.06 (1.01–1.11), .0153
Fitting model by two-piecewise logistic regression	
Inflection point	47.32
<47.32	1.07 (1.02–1.12), .0076
>47.32	1.26 (1.08–1.46), .0028
*P* for log likely ratio test	.021

Adjusted for all covariates presented in Table 2.

CI = confidence interval, OR = odds ratio, RFM = relative fat mass.

Together, these findings support a primarily linear, positive association between RFM and cognitive impairment in this cross-sectional analysis, with exploratory evidence suggesting a possible acceleration at higher adiposity levels. Given the NHANES design, all dose–response patterns are descriptive and should not be interpreted as causal.

### 3.4. Subgroup analyses

**Figure 3.**
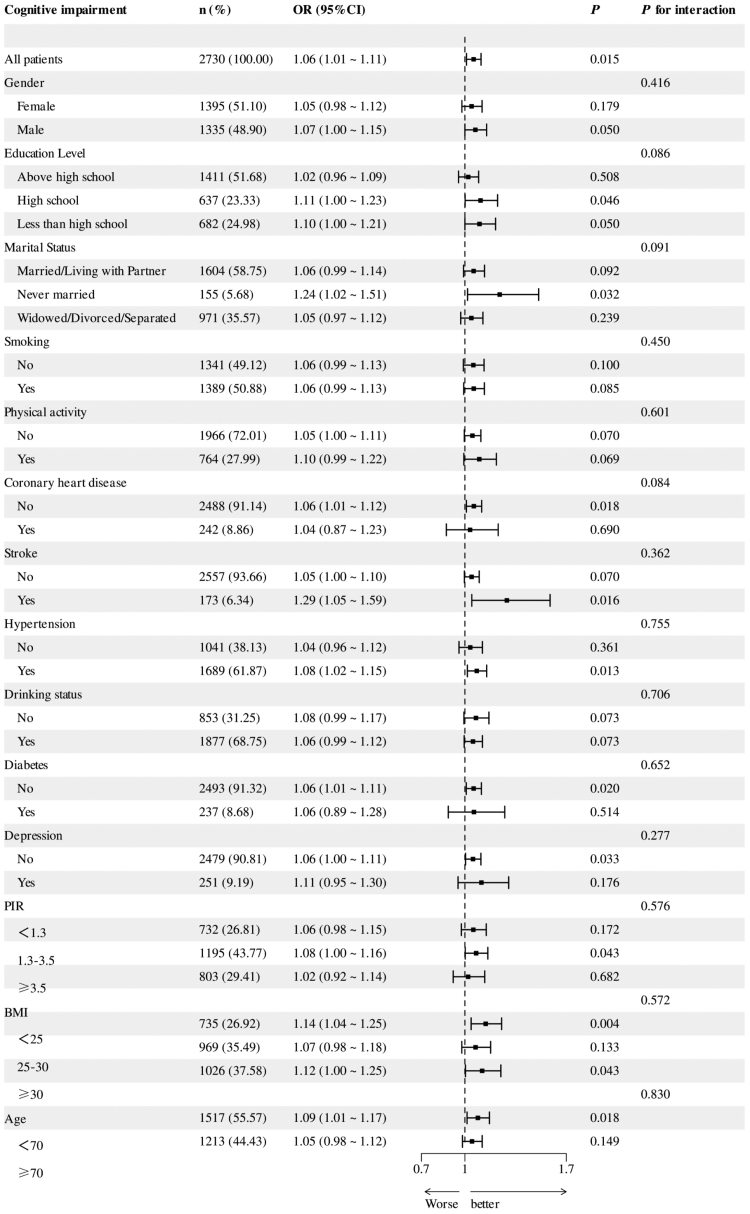
Subgroup analysis for the association between RFM and cognitive impairment. Adjust for gender, age, race, education level, marital status, PIR, smoking, physical activity, coronary heart disease, stroke, total energy intake, hypertension, BMI, drinking status, diabetes, and depression. BMI = body mass index, CI = confidence interval, OR = odds ratio, PIR = poverty–income ratio, RFM = relative fat mass.

Figure 3 shows broadly consistent, positive cross-sectional associations between RFM and cognitive impairment across strata, with most interaction tests null. The only clear heterogeneity was by age (*P* for interaction ≈ .02): the association was stronger among adults < 70 years (OR ≈ 1.09; 95% CI ~1.01–1.17) and attenuated in those ≥ 70 years (OR ≈ 1.05; 95% CI ~0.98–1.12). Within strata (exploratory), several groups showed statistically significant positive associations: participants with prior stroke (OR ≈ 1.29; 95% CI ~1.05–1.59), those with hypertension (OR ≈ 1.08; 95% CI ~1.02–1.15), and BMI < 25 (OR ≈ 1.14; 95% CI ~1.04–1.25) or ≥ 30 (OR ≈ 1.12; 95% CI ~1.00–1.25). Modest associations were also observed among married/partnered and never-married participants. In contrast, there was no meaningful heterogeneity by sex, education, smoking, alcohol use, diabetes, CHD, depression, physical activity, or PIR (all *P* for interaction > .05).

Taken together, subgroup results support a generally homogeneous, positive association between RFM and cognitive impairment, with stronger effects in younger older adults and at the lower and higher ends of BMI. Given multiple comparisons and the cross-sectional design, these findings are descriptive and hypothesis-generating.

### 3.5. Mediation analysis

Mediation analysis identified hypertension, physical activity, and depression as potential mediators in the association between RFM and cognitive impairment after adjustment for covariates. In single-mediator models, the ACME was statistically significant for hypertension (ACME ≈ 0.0040, *P* = .013) and physical activity (ACME ≈ 0.0038, *P* = .027), while the ACME for depression was small and not statistically significant (ACME ≈ 0.0023, *P* = .360). Diabetes showed a negligible, nonsignificant mediation effect (ACME≈−0.0001, *P* = .947). Notably, BMI exerted a negative (inhibitory) mediation effect (ACME≈−0.0486, *P* = .060), suggesting that BMI may partially offset the association between RFM and cognitive impairment (see Supplementary Fig. S2, Supplemental Digital Content 4). Proportions mediated in the single-mediator models were approximately 10.0% for hypertension and 9.4% for physical activity status, respectively.

## 4. Discussion

In this cross-sectional analysis of nationally representative NHANES 2011–2014 data, higher RFM was associated with greater odds of cognitive impairment among US older adults after multivariable adjustment. The association was generally linear and remained directionally consistent in sensitivity analyses, although the cross-sectional design precludes causal inference. These findings suggest that RFM, a simple index derived from height and WC, may capture adiposity-related metabolic and vascular burden that is relevant to cognitive health. The partial mediation by hypertension and physical activity status further supports the potential importance of vascular and lifestyle pathways linking excess adiposity to cognitive vulnerability in later life. Clinically, RFM may serve as a scalable adjunct to conventional anthropometric measures for identifying older adults who may benefit from more comprehensive cardiometabolic and cognitive risk assessment.

This analysis is among the first cross-sectional studies to examine the RFM–cognitive impairment relationship in nationally representative NHANES data. Prior evidence supports biological plausibility: RFM, a surrogate for overall and centrally distributed adiposity, relates to metabolic derangement and conditions tied to cognitive decline, including hypertension, insulin resistance, dyslipidemia, and carotid atherosclerosis.^[14,15]^ Excess visceral fat promotes low-grade systemic inflammation (e.g., elevated interleukin-6 and tumor necrosis factor-α) that can traverse the blood–brain barrier and perturb neuronal signaling.^[16,17]^ Consistent with these intersections, higher RFM has been associated with arterial stiffness and reduced cerebral perfusion, while oxidative stress and endothelial dysfunction foster microvascular injury implicated in cardiovascular and cognitive disorders.^[18]^ In our data, hypertension and physical activity status partially mediated the RFM–cognition association, supporting the relevance of vascular and lifestyle pathways, whereas depression and diabetes showed minimal mediation. BMI exhibited small suppressive mediation, underscoring that conventional anthropometrics may underrepresent metabolically active fat captured by RFM.^[11,19]^

The international literature suggests that the relationship between adiposity and cognition is not uniform across populations. Studies from China and other population-based cohorts have reported U-shaped, inverse, or otherwise heterogeneous associations, which may reflect differences in age structure, ethnicity, nutritional status, sarcopenia, vascular burden, adiposity indices, and cognitive assessments. These differences highlight the need to evaluate adiposity–cognition associations across diverse populations and with multiple anthropometric indicators.^[20,21]^ Because BMI cannot distinguish fat from lean mass, whereas RFM incorporates height and central fat distribution, RFM may better track risk. Overall, RFM emerges as a simple, cost-effective adjunct to identify older adults at elevated neurovascular risk and to complement BMI/WC alongside routine cognitive screening.

Several factors may explain why our study observed a positive association between RFM and cognitive impairment despite prior reports of inverse or U-shaped associations between adiposity and cognition. First, many previous studies relied primarily on BMI, which does not distinguish fat mass from lean mass and may therefore misclassify older adults with sarcopenia, frailty, or unintentional weight loss. In late life, lower BMI may reflect underlying illness, muscle loss, or prodromal neurodegeneration rather than a truly protective adiposity profile, contributing to an apparent “obesity paradox.” In contrast, RFM incorporates WC and height and may better capture central adiposity and residual metabolic risk beyond BMI. Second, the positive association in our study emerged after comprehensive adjustment, including BMI, suggesting that higher RFM may reflect the adverse component of adiposity not adequately captured by conventional anthropometric measures. Third, RFM is more closely related to central fat accumulation, which is strongly linked to hypertension, inflammation, endothelial dysfunction, and cerebrovascular injury. These mechanisms are biologically consistent with cognitive vulnerability in older adults. Therefore, our findings do not necessarily contradict previous inverse or U-shaped findings; rather, they suggest that the direction of the adiposity–cognition association may depend on the adiposity index used, the degree of adjustment for confounding, and the extent to which the measure captures central adiposity versus general body size.

Higher RFM may influence cognitive function through several interrelated biological pathways. First, excess adiposity can promote chronic low-grade systemic inflammation. Proinflammatory cytokines may impair blood–brain barrier integrity, activate neuroinflammatory responses, and disrupt neuronal homeostasis, thereby linking adiposity-related metabolic stress to cognitive vulnerability in later life.^[16]^

Second, metabolic and vascular mechanisms may also contribute to the observed association. Adiposity-related insulin resistance and dyslipidemia can impair neuronal insulin signaling, cerebral glucose metabolism, and lipid-mediated endothelial function.^[22,23]^ In parallel, central adiposity is closely related to elevated blood pressure and endothelial dysfunction, which may promote cerebral small-vessel disease, white-matter injury, and chronic hypoperfusion. These pathways are consistent with the partial mediation by hypertension observed in our analysis.^[24,25]^

Third, oxidative stress, adverse adipokine profiles, and physical inactivity may further amplify adiposity-related neurovascular risk. Leptin-related inflammatory signaling and adipokine imbalance may contribute to mitochondrial stress and endothelial inflammation.^[17,26]^ Low physical activity may reduce brain-derived neurotrophic factor, impair cerebrovascular reactivity, and limit neuroplasticity.^[27,28]^ Together, these inflammatory, metabolic, vascular, and behavioral pathways provide a biologically coherent explanation for the positive association between RFM and cognitive impairment, while the cross-sectional design precludes causal inference.

Potential reverse causation must also be considered. Prodromal cognitive decline can alter appetite, physical activity, medication adherence, and body composition – leading to inactivity-related fat gain or weight loss with sarcopenia – and thereby bias cross-sectional associations.^[29,30]^ Executive dysfunction may impede hypertension control and reduce activity, indirectly increasing adiposity and vascular burden.^[31]^ Conversely, late-life weight loss may precede dementia, so lower adiposity can reflect underlying neurodegeneration rather than confer protection, obscuring dose–response gradients.^[32]^ Differential misclassification – e.g., BMI underdetection sarcopenic obesity, while RFM emphasizes central fat – may further bias estimates toward nonlinearity or the null.^[33,34]^ Longitudinal studies with repeated assessments of adiposity, behavior, and cognition are required to clarify temporality and causal pathways.

Subgroup analyses indicated that the positive association between RFM and cognitive impairment was broadly consistent across strata, with only limited evidence for effect modification. Age and hypertension modestly altered the magnitude of association: estimates were larger among adults < 70 years and attenuated in those ≥ 70 years; higher odds were also observed in participants with prior stroke or hypertension, and signals emerged at both low and high BMI categories, suggesting vulnerability related to vascular load and body-composition extremes. These patterns are compatible with literature linking adiposity to carotid atherosclerosis and endothelial dysfunction^[35]^ and align with frameworks emphasizing lifestyle and vascular pathways in late-life cognition.^[36]^ Mechanistically, hypertension-related small-vessel disease, impaired neurovascular coupling, and age-related reductions in physiological resilience are plausible contributors.^[37]^ Absence of clear heterogeneity by sex, education, diabetes, depression, and lifestyle factors should be interpreted cautiously, given multiple testing and limited power for some subgroups. Overall, these subgroup findings are hypothesis-generating and require longitudinal confirmation to distinguish true modifiers (e.g., age, hypertension, and prior cerebrovascular disease) from residual confounding.

From a translational perspective, RFM, calculated from height and WC, provides a simple and scalable adjunct for risk stratification in older adults. It should complement, rather than replace, BMI, WC, and routine cognitive screening. The observed partial mediation by hypertension and physical inactivity suggests that blood pressure control and physical activity may be relevant areas for future longitudinal and intervention research on adiposity-related vulnerability to cognitive impairment.

This study has several strengths. First, it used nationally representative NHANES data, allowing the findings to reflect community-dwelling older adults in the United States. Second, all analyses incorporated NHANES survey weights, strata, and primary sampling units, thereby accounting for the complex multistage sampling design. Third, cognitive impairment was defined using multiple cognitive assessments covering memory, verbal fluency, processing speed, attention, and working memory rather than a single test. Finally, the analyses included extensive adjustment for demographic, socioeconomic, lifestyle, dietary, anthropometric, and clinical covariates, and the robustness of the findings was evaluated through sensitivity, subgroup, dose–response, and mediation analyses.

Limitations: This study has several important limitations. First, it uses a cross-sectional NHANES 2011–2014 sample, which precludes establishing temporality or causal inference and leaves open the possibility of bidirectional relationships between adiposity and cognition; all dose–response patterns should therefore be interpreted as descriptive rather than causal. Because NHANES includes civilian, noninstitutionalized adults, our findings may not be generalizable to older adults living in nursing homes, long-term care facilities, or other institutional settings. Second, cognitive impairment was operationalized from 3 brief instruments (CERAD learning/recall, animal fluency, and DSST) using education-specific 25th-percentile cutoffs; while consistent with contemporary NHANES practice, this approach may misclassify borderline cases and underdetect deficits in domains not captured by these tests. Third, despite survey weighting and extensive covariate adjustment, residual and unmeasured confounding cannot be excluded; moreover, missing covariates were handled via complete-case analysis, which can introduce selection bias and reduce generalizability. Finally, the RCS and two-piecewise models were exploratory; precision diminished in the upper tail and the apparent inflection at higher RFM should be verified in longitudinal settings.

## 5. Conclusion

In a nationally representative sample of US older adults from NHANES 2011–2014, higher RFM was associated with greater odds of test-based cognitive impairment after multivariable adjustment. Dose–response analyses suggested a largely linear association, while subgroup and mediation findings were exploratory and hypothesis-generating. The observed mediation patterns involving hypertension and physical activity status suggest that vascular and lifestyle factors may be relevant to the RFM–cognition association, but these findings should not be interpreted as evidence of causal or interventional effects. Given the cross-sectional design, residual confounding, and possible reverse causation, longitudinal studies with repeated adiposity and cognitive assessments are needed to clarify temporality, causal pathways, and the potential clinical utility of RFM in cognitive risk assessment.

## Acknowledgments

We thank the staff of the National Center for Health Statistics and the Centers for Disease Control and Prevention for designing, collecting, and maintaining the NHANES data. No individuals are named in this Acknowledgments section.

## Author contributions

**Conceptualization:** Zhihui Wu.

**Investigation:** Weijiao Feng.

**Methodology:** Lili Zhang.

**Supervision:** Lili Zhang, Weijiao Feng.

**Validation:** Weijiao Feng, Yan Huang.

**Writing – original draft:** Zhihui Wu, Yan Huang.

**Writing – review & editing:** Yan Huang.









## References

[1] Livingston G Huntley J Liu KY. Dementia prevention, intervention, and care: 2024 report of the Lancet standing Commission. Lancet. 2024;404:572–628. 39096926 10.1016/S0140-6736(24)01296-0

[2] Salari N Lotfi F Abdolmaleki A. The global prevalence of mild cognitive impairment in geriatric population with emphasis on influential factors: a systematic review and meta-analysis. BMC Geriatr. 2025;25:313. 40329163 10.1186/s12877-025-05967-w PMC12053864

[3] Chen P Cai H Bai W. Global prevalence of mild cognitive impairment among older adults living in nursing homes: a meta-analysis and systematic review of epidemiological surveys. Transl Psychiatry. 2023;13:88. 36906613 10.1038/s41398-023-02361-1 PMC10008549

[4] Öksüz N Ghouri R Taşdelen B Uludüz D Özge A. Mild cognitive impairment progression and Alzheimer’s disease risk: a comprehensive analysis of 3553 cases over 203 months. J Clin Med. 2024;13:518. 38256652 10.3390/jcm13020518 PMC10817043

[5] Tahami Monfared AA Byrnes MJ White LA Zhang Q. The humanistic and economic burden of Alzheimer’s disease. Neurol Ther. 2022;11:525–51. 35192176 10.1007/s40120-022-00335-x PMC9095804

[6] Liao X Li Y Zhang Z. Associations of the body roundness index with cognitive function in US older adults and the mediating role of depression: a cross-sectional study from the NHANES 2011-2014. Sci Rep. 2025;15:16884. 40374704 10.1038/s41598-025-01383-7 PMC12081760

[7] Sweatt K Garvey WT Martins C. Strengths and limitations of BMI in the diagnosis of obesity: what is the path forward? Curr Obes Rep. 2024;13:584–95. 38958869 10.1007/s13679-024-00580-1 PMC11306271

[8] Wang Z Li W Lv L. Association between relative fat mass (RFM) and chronic kidney disease (CKD): data from NHANES 2005-2018. Sci Rep. 2025;15:25673. 40664977 10.1038/s41598-025-09334-y PMC12264183

[9] Baradaran H Gupta A. Carotid artery stiffness: imaging techniques and impact on cerebrovascular disease. Front Cardiovasc Med. 2022;9:852173. 35369341 10.3389/fcvm.2022.852173 PMC8964780

[10] Zheng Y Huang C Jin J Zhao Y Cui H Wei C. Association between stroke and relative fat mass: a cross-sectional study based on NHANES. Lipids Health Dis. 2024;23:354. 39482681 10.1186/s12944-024-02351-2 PMC11526522

[11] Zwartkruis VW Suthahar N Idema DL. Relative fat mass and prediction of incident atrial fibrillation, heart failure and coronary artery disease in the general population. Int J Obes (Lond). 2023;47:1256–62. 37684330 10.1038/s41366-023-01380-8

[12] Dong X Li S Chen J Li Y Wu Y Zhang D. Association of dietary ω-3 and ω-6 fatty acids intake with cognitive performance in older adults: National Health and Nutrition Examination Survey (NHANES) 2011-2014. Nutr J. 2020;19:25. Published 2020 Mar 28. 32222145 10.1186/s12937-020-00547-7 PMC7103069

[13] Liu C He L Li Y Yang A Zhang K Luo B. Diabetes risk among US adults with different socioeconomic status and behavioral lifestyles: evidence from the National Health and Nutrition Examination Survey. Front Public Health. 2023;11:1197947. 37674682 10.3389/fpubh.2023.1197947 PMC10477368

[14] Adeva-Andany MM Domínguez-Montero A Adeva-Contreras L Fernández-Fernández C Carneiro-Freire N González-Lucán M. Body fat distribution contributes to defining the relationship between insulin resistance and obesity in human diseases. Curr Diabetes Rev. 2024;20:e160823219824. 37587805 10.2174/1573399820666230816111624

[15] Pérez-García A Torrecilla-Parra M Fernández-de Frutos M Martín-Martín Y Pardo-Marqués V Ramírez CM. Posttranscriptional regulation of insulin resistance: implications for metabolic diseases. Biomolecules. 2022;12:208. 35204710 10.3390/biom12020208 PMC8961590

[16] Versele R Sevin E Gosselet F Fenart L Candela P. TNF-α and IL-1β modulate blood-brain barrier permeability and decrease amyloid-β peptide efflux in a human blood-brain barrier model. Int J Mol Sci. 2022;23:10235. 36142143 10.3390/ijms231810235 PMC9499506

[17] Liu X Zheng H. Modulation of Sirt1 and FoxO1 on hypothalamic leptin-mediated sympathetic activation and inflammation in diet-induced obese rats. J Am Heart Assoc. 2021;10:e020667. 34259031 10.1161/JAHA.120.020667 PMC8483493

[18] Liu W Chen Z Ortega D. Arterial elasticity, endothelial function and intracranial vascular health: a multimodal MRI study. J Cereb Blood Flow Metab. 2021;41:1390–7. 33081567 10.1177/0271678X20956950 PMC8142128

[19] Shen W Cai L Wang B Wang Y Wang N Lu Y. Associations of relative fat mass, a novel adiposity indicator, with non-alcoholic fatty liver disease and cardiovascular disease: data from SPECT-China. Diabetes Metab Syndr Obes. 2023;16:2377–87. 37577042 10.2147/DMSO.S423272 PMC10422986

[20] Liang Z Jin W Huang L Chen H. Body mass index, waist circumference, hip circumference, abdominal volume index, and cognitive function in older Chinese people: a nationwide study. BMC Geriatr. 2024;24:925. 39516791 10.1186/s12877-024-05521-0 PMC11546056

[21] Booranasuksakul U Macdonald IA Stephan BCM Siervo M. Body composition, sarcopenic obesity, and cognitive function in older adults: findings from the National Health and Nutrition Examination Survey (NHANES) 1999-2002 and 2011-2014. J Am Nutr Assoc. 2024;43:539–52. 38564377 10.1080/27697061.2024.2333310

[22] Jang MH Song J. Adenosine and adenosine receptors in metabolic imbalance-related neurological issues. Biomed Pharmacother. 2024;177:116996. 38897158 10.1016/j.biopha.2024.116996 PMC12021433

[23] Yamamoto M Guo DH Hernandez CM Stranahan AM. Endothelial Adora2a activation promotes blood-brain barrier breakdown and cognitive impairment in mice with diet-induced insulin resistance. J Neurosci. 2019;39:4179–92. 30886019 10.1523/JNEUROSCI.2506-18.2019 PMC6529868

[24] Webb AJS Birks JS Feakins KA. Cerebrovascular effects of sildenafil in small vessel disease: the OxHARP trial. Circ Res. 2024;135:320–31. 38832504 10.1161/CIRCRESAHA.124.324327 PMC11227301

[25] Ungvari Z Toth P Tarantini S. Hypertension-induced cognitive impairment: from pathophysiology to public health. Nat Rev Nephrol. 2021;17:639–54. 34127835 10.1038/s41581-021-00430-6 PMC8202227

[26] Frühbeck G Catalán V Rodríguez A. Involvement of the leptin-adiponectin axis in inflammation and oxidative stress in the metabolic syndrome. Sci Rep. 2017;7:6619. 28747790 10.1038/s41598-017-06997-0 PMC5529549

[27] Oyovwi MO Ogenma UT Onyenweny A. Exploring the impact of exercise-induced BDNF on neuroplasticity in neurodegenerative and neuropsychiatric conditions. Mol Biol Rep. 2025;52:140. 39832087 10.1007/s11033-025-10248-1

[28] Xue B Waseem SMA Zhu Z. Brain-derived neurotrophic factor: a connecting link between nutrition, lifestyle, and Alzheimer’s disease. Front Neurosci. 2022;16:925991. 35692417 10.3389/fnins.2022.925991 PMC9177140

[29] Kimura A Sugimoto T Niida S Toba K Sakurai T. Association between appetite and sarcopenia in patients with mild cognitive impairment and early-stage Alzheimer’s disease: a case-control study. Front Nutr. 2018;5:128. 30619874 10.3389/fnut.2018.00128 PMC6305366

[30] Tou NX Wee SL Pang BWJ. Associations of fat mass and muscle function but not lean mass with cognitive impairment: the Yishun Study. PLoS One. 2021;16:e0256702. 34437646 10.1371/journal.pone.0256702 PMC8389410

[31] Hung TH Chen VC Chuang YC. Investigating the effect of hypertension on vascular cognitive impairment by using the resting-state functional connectome. Sci Rep. 2024;14:4580. 38403657 10.1038/s41598-024-54996-9 PMC10894879

[32] Cui C Mackey RH Shaaban CE Kuller LH Lopez OL Sekikawa A. Associations of body composition with incident dementia in older adults: Cardiovascular Health Study-Cognition Study. Alzheimer’s Dement. 2020;16:1402–11. 32803916 10.1002/alz.12125 PMC7881417

[33] Paek JK Kim J Kim K Lee SY. Usefulness of relative fat mass in estimating body adiposity in Korean adult population. Endocr J. 2019;66:723–9. 31142689 10.1507/endocrj.EJ19-0064

[34] Orwoll ES Blackwell T Cummings SR. CT muscle density, D3Cr muscle mass, and body fat associations with physical performance, mobility outcomes, and mortality risk in older men. J Gerontol A Biol Sci Med Sci. 2022;77:790–9. 34529767 10.1093/gerona/glab266 PMC8974337

[35] Louka AM Sagris D Ntaios G. Immunity, vascular aging and stroke. Curr Med Chem. 2022;29:5510–21. 34979888 10.2174/0929867329666220103101700

[36] Li M Qian M Kyler K Xu J. Adipose tissue-endothelial cell interactions in obesity-induced endothelial dysfunction. Front Cardiovasc Med. 2021;8:681581. 34277732 10.3389/fcvm.2021.681581 PMC8282205

[37] Stankovics L Ungvari A Fekete M. The vasoprotective role of IGF-1 signaling in the cerebral microcirculation: prevention of cerebral microhemorrhages in aging. Geroscience. 2025;47:445–55. 39271571 10.1007/s11357-024-01343-5 PMC11872839

