# Supplementary material for: Association between relative fat mass and cognitive impairment in older adults: A cross-sectional study using NHANES 2011–2014 data
Source: Medicine (Baltimore). 2026 Jul 10;105(28):e49621. doi: 10.1097/MD.0000000000049621 (PMC13362864; doi:10.1097/MD.0000000000049621)
Supplement: Supplementary file 1 [file medi-105-e49621-s001.docx]

Supplementary Table S2: Multicollinearity check of predictors used in the RFM–cognitive impairment analysis

| Variable | VIF | Tolerance (1/VIF) |
| --- | --- | --- |
| PIR | 1.297355923936149 | 0.7707984998950961 |
| Gender | 1.2920457346571963 | 0.7739664109222253 |
| Drinking status | 1.2667374641287812 | 0.7894295608346642 |
| Education Level | 1.255070501811737 | 0.7967679891738878 |
| Smoking | 1.201574993632803 | 0.8322410214086032 |
| Total Energy Intake | 1.1265753212404397 | 0.8876459311206363 |
| Marital Status | 1.1261493176250006 | 0.8879817128593178 |
| BMI | 1.0993337920848445 | 0.9096418278051276 |
| Age | 1.0944436603574859 | 0.9137062383580009 |
| Hypertension | 1.0920651378019839 | 0.9156962944652873 |
| Depression | 1.0761496383376472 | 0.929238801348038 |
| Race | 1.073845094812533 | 0.9312330100782137 |
| Coronary heart disease | 1.0548998550867295 | 0.9479572825590958 |
| Stroke | 1.0387381766240276 | 0.9627065053583287 |
| Physical activity | 1.0226880718466698 | 0.9778152571920568 |
| Diabetes | 1.0023785656493842 | 0.997627078500184 |
