# Supplementary material for: Association between relative fat mass and cognitive impairment in older adults: A cross-sectional study using NHANES 2011–2014 data
Source: Medicine (Baltimore). 2026 Jul 10;105(28):e49621. doi: 10.1097/MD.0000000000049621 (PMC13362864; doi:10.1097/MD.0000000000049621)
Supplement: Supplementary file 2 [file medi-105-e49621-s002.docx]

**Supplementary Table S1:** Sensitivity analyses of the association between relative fat mass and cognitive impairment

| Analysis | OR | LCL | UCL | N |
| --- | --- | --- | --- | --- |
| Base adjusted | 1.009216 | 0.976711 | 1.042803 | 2730 |
| Main adjusted | 1.05146 | 1.019292 | 1.087419 | 2730 |
| Exclude BMI ≥95th pct | 1.104837 | 1.036484 | 1.177699 | 2592 |
| Exclude RFM outliers (1–99%) | 1.098708 | 1.026743 | 1.175717 | 2674 |
| Log-transformed RFM | 6.274713 | 1.244199 | 31.644458 | 2730 |
| Waist circumference (z-score) | 1.373676 | 1.023355 | 1.84392 | 2730 |
