# Supplementary figures and images for: Association between relative fat mass and cognitive impairment in older adults: A cross-sectional study using NHANES 2011–2014 data
Source: Medicine (Baltimore). 2026 Jul 10;105(28):e49621. doi: 10.1097/MD.0000000000049621 (PMC13362864; doi:10.1097/MD.0000000000049621)

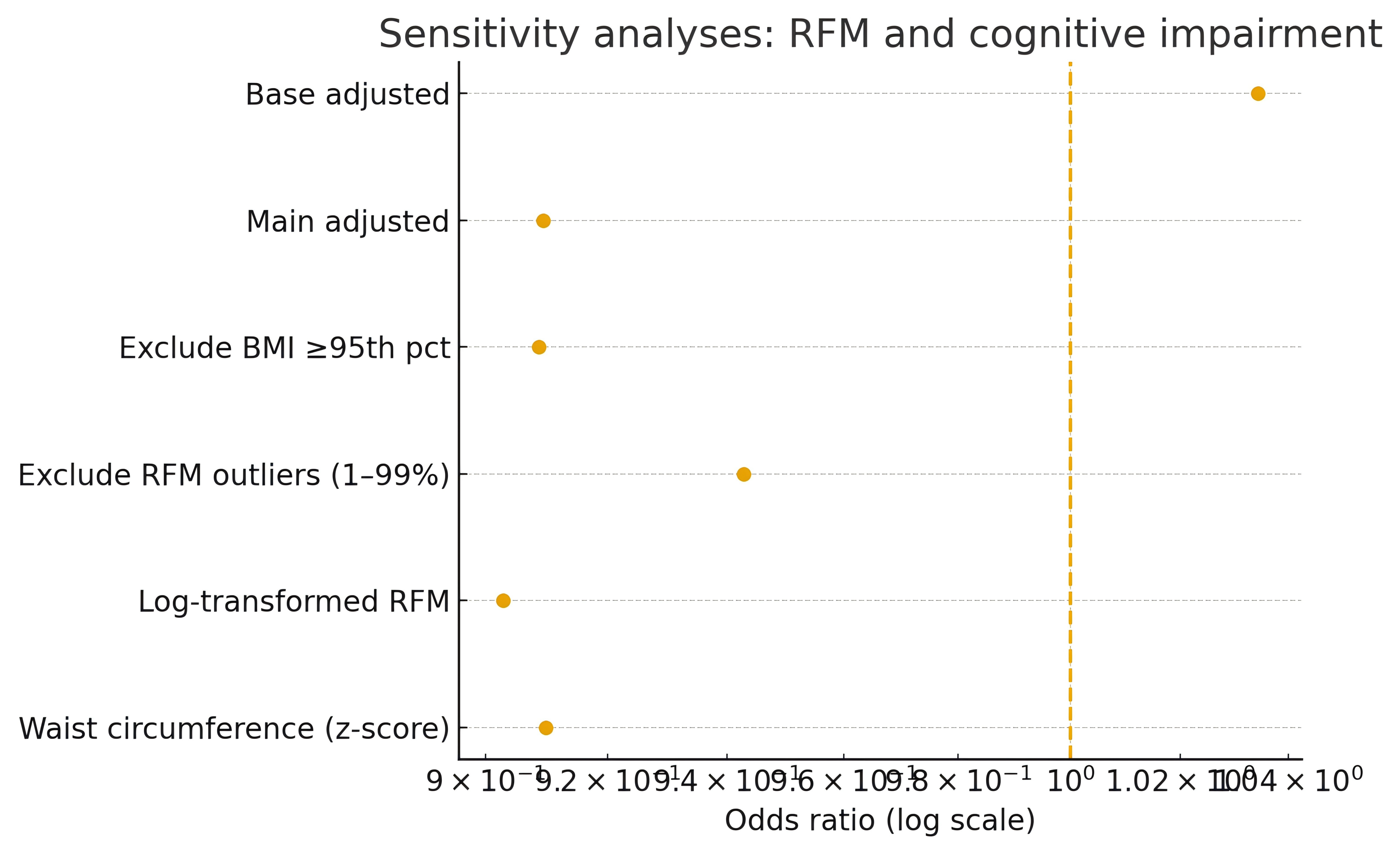

Supplement: Supplementary file 3 [file medi-105-e49621-s003.tiff]

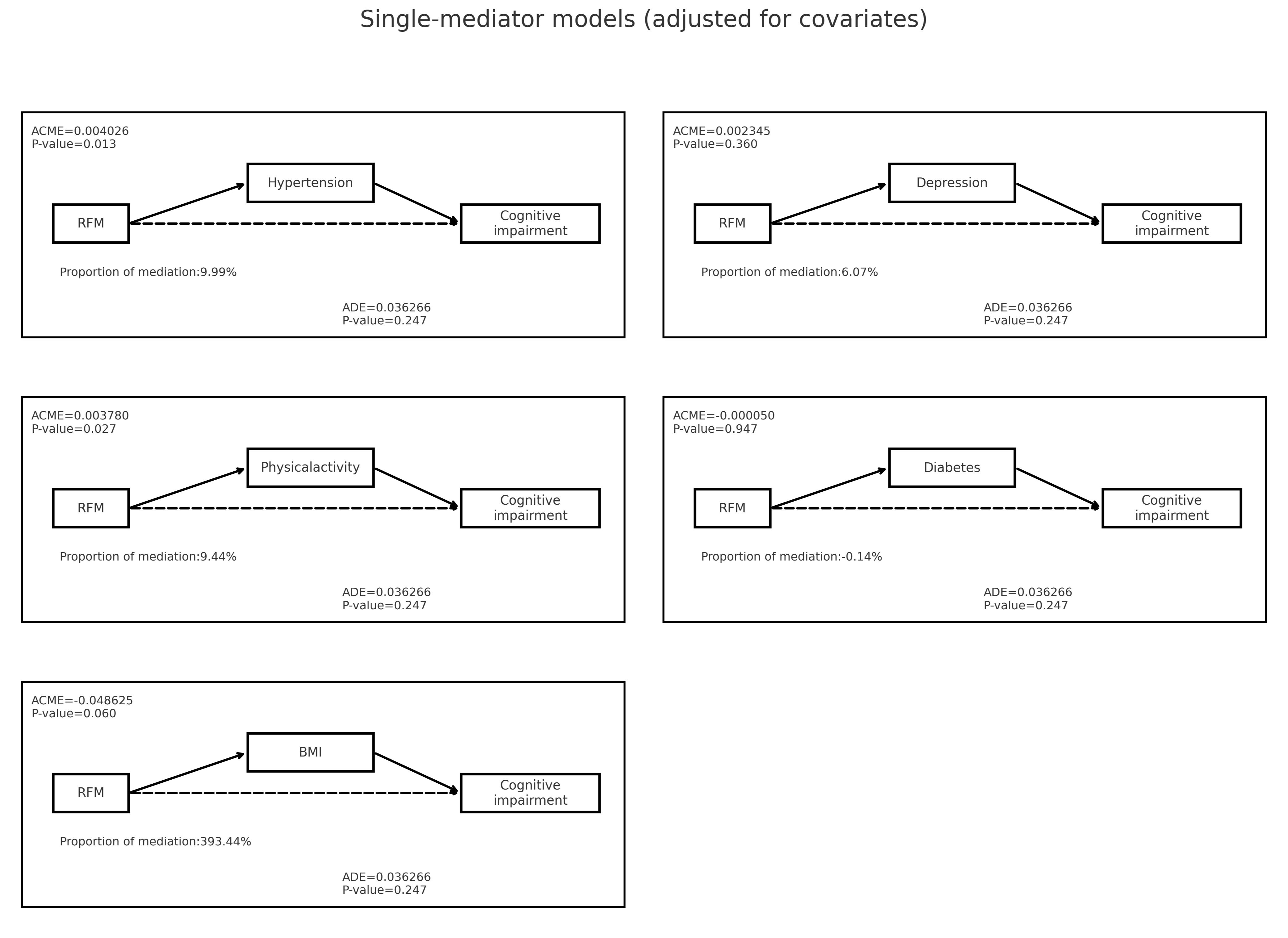

Supplement: Supplementary file 4 [file medi-105-e49621-s004.tif]
